# Integrated transcriptomic and metabolomic analysis of the antibacterial mechanism of *Rhizoma Coptidis* extract against *Staphylococcus epidermidis* ATCC 35984

**DOI:** 10.1186/s12866-025-04169-z

**Published:** 2025-08-05

**Authors:** Lizhuang Wang, Yan Xing, Shuai Yang, Huan Zhang, Laiji Ma, Li Shao

**Affiliations:** 1 School of Perfume and Aroma Technology, Shanghai Institute of Technology, Shanghai, 201418 P. R. China; 2 Shandong Academy of Pharmaceutical Sciences, Jinan, Shandong 250098 P. R. China

**Keywords:** *Rhizoma Coptidis* extract, *Staphylococcus epidermidis*, Antibacterial activity, Biofilm, Transcriptomic, Metabolomic

## Abstract

**Background:**

Biofilm formation is a key virulence factor in *Staphylococcus epidermidis*. *Rhizoma Coptidis*, the medicinal root of *Coptis chinensis*, has been traditionally used in Chinese medicine for its broad-spectrum antimicrobial properties.

**Results:**

This study investigated the antibacterial and anti-biofilm effects of *Rhizoma Coptidis* extract against *S. epidermidis* ATCC 35984. UPLC-MS/MS analysis revealed the chemical composition of the extract. The extract significantly reduced ATPase and succinate dehydrogenase activities, decreased membrane potential, and disrupted cell wall and membrane integrity. These effects led to increased extracellular alkaline phosphatase activity and leakage of proteins and nucleic acids. Anti-biofilm activity was further confirmed using scanning electron microscopy and confocal laser scanning microscopy. Transcriptomic and metabolomic analyses identified significant changes in 366 genes and 286 metabolites after treatment. Integrated omics analysis suggested that the extract impaired cell membrane and wall integrity, disrupted amino acid and nucleic acid metabolism, inhibited the TCA cycle, reduced nitrate reductase activity, suppressed efflux systems, and hindered biofilm formation.

**Conclusions:**

The study provides new insights into the antibacterial and anti-biofilm mechanisms of *Rhizoma Coptidis* extract against *S. epidermidis*, highlighting its potential as a therapeutic agent for combating biofilm-associated infections.

**Supplementary Information:**

The online version contains supplementary material available at 10.1186/s12866-025-04169-z.

## Background

*Staphylococcus epidermidis* is a coagulase-negative *staphylococcus* and a normal constituent of the mucosal and skin microbiota in humans and other mammals. It can be isolated from all skin microenvironments, including neutral, dry, moist, and oily skin areas [1]. While *S. epidermidis* is generally regarded as a commensal organism on the skin, an increasing body of evidence suggests that alterations in its abundance under certain conditions may become detrimental to the host [2]. Changes in the environmental factors supporting skin microbiota, whether through anthropogenic or natural disruptions, can significantly shift the behavior of *S. epidermidis* from a benign symbiont to a pathogenic organism [3]. When *S. epidermidis* is stressed or injured, it forms biofilms that can resist the bactericidal effects of antibiotics, facilitating a self-protection mechanism and preventing damage from external factors. Biofilm formation is considered one of the most important virulence factors of *S. epidermidis*. Additionally, *S. epidermidis* induces the production of inflammatory cytokines, such as Tumor Necrosis Factor-α, Interleukin-1β, and Interleukin-6, from surrounding cells [4]. It can also exacerbate inflammation by activating Toll-like receptor 2 and secreting esterases that disrupt the hair follicle wall [5]. Furthermore, *S. epidermidis* has been shown to induce the migration of T-helper 17 cells (Th17) to the epidermis. Th17 cells are commonly found in conditions like squamous cell carcinoma, actinic keratosis, and psoriasis, suggesting that *S. epidermidis* may play a role in promoting squamous cell carcinoma pathogenesis [6, 7]. Moreover, numerous studies have reported correlations between the abundance of *S. epidermidis* and various inflammatory skin diseases, including dandruff [8, 9], seborrheic dermatitis [10], and rosacea [11].

*Coptis chinensis*, first documented in the *Shennong Bencao Jing* (Divine Farmer’s Materia Medica), is an herbaceous plant belonging to the *Ranunculaceae* family. It is also known by other names, including “jizhua lian,” “chuan lian,” and “wei lian.” This plant is primarily distributed in regions of China such as Hubei, Sichuan, and Yunnan province. The rhizome of *Coptis chinensis*, referred to as *Rhizoma Coptidis*, is the medicinal part used in traditional Chinese medicine. The chemical composition of *Rhizoma Coptidis* is complex, with identified components including alkaloids, lignans, flavonoids, organic acids, and essential oils. Natural plant extracts have been used for antibacterial and anti-biofilm purposes [12, 13]. Park et al. [14] reported that treatment with *Rhizoma Coptidis* and *Pelargonium sidoides* significantly reduced the protein expression levels of lipopolysaccharide (LPS)-induced nuclear factor kappa B (NF-κB) and phosphorylated inhibitor of NF-κB in RAW264.7 macrophages. Research has also shown that berberine, a major alkaloid in *Rhizoma Coptidis*, reduces the protein expression of phosphoenolpyruvate carboxykinase and glucose-6-phosphatase in liver tissue, improves glucose tolerance, and decreases plasma hyperlipidemia [15]. Zhang et al. [16] observed a synergistic effect against multidrug-resistant *S. epidermidis* when berberine was combined with commonly used antibiotics, such as penicillin, lincomycin, and amoxicillin. Some studies have also shown that berberine at concentrations greater than 30 µg/mL is sufficient to exhibit antimicrobial activity against *S. epidermidis* and significantly inhibit biofilm formation [17, 18]. Studies have also confirmed that extracts of *Rhizoma Coptidis* exhibit broad-spectrum antimicrobial activity. Li et al. [19] demonstrated that aqueous extracts of *Rhizoma Coptidis* significantly inhibited the formation of biofilms by *Streptococcus suis*, with notable changes in adhesion-related genes such as *gapdh*, *sly*, and *mrp*. Furthermore, recent study has indicated the combined application of *Rosa rugosa Thunb.* and *Rhizoma Coptidis* significantly affected the integrity of the *Malassezia furfur* cell membrane and potentially modulated its formation processes, and exhibited synergistic antifungal effects [20]. Additionally, the *Rhizoma Coptidis* extract (RCE) has been shown to possess potent antibacterial and bactericidal activity against *Streptococcus pneumoniae* and can effectively inhibit its biofilm formation [21].

RCE exhibits broad-spectrum antimicrobial activity against various bacteria and fungi, and, compared to antibiotics, it is less prone to inducing resistance [22, 23]. However, most current research on the antimicrobial properties of RCE has focused primarily on foodborne pathogens, with applications largely centered on food preservation and antimicrobial protection. There is limited research on its effects against skin microbiota, and the underlying mechanisms of its antimicrobial activity in this context remain inadequately explored.

In this study, we analyzed the chemical composition of RCE using UPLC-MS/MS and evaluated its effects on the growth, energy metabolism, cell wall and cell membrane integrity, and biofilm formation of *S. epidermidis* ATCC 35984 (*S. epidermidis* 35984) through in vitro experiments. By integrating transcriptomic and metabolomic analysis, we systematically investigated the antibacterial activity and biofilm inhibition mechanisms of RCE against *S. epidermidis* 35984. In addition to its antimicrobial and anti-biofilm properties, the findings from this study had important implications for the potential application of RCE in cosmetic formulations. Given the growing demand for natural antimicrobial agents in skincare products, RCE offered a promising alternative due to its ability to inhibit biofilm formation. This study highlighted the potential of RCE as an effective ingredient for cosmetic products, providing a scientific basis for its use in combating skin-related infections.

## Materials and methods

### Reagents, bacterial strains and culture conditions

Rhodamine 123 Solution: Dissolve 0.1 g of Rhodamine 123 in phosphate-buffered saline (PBS, 0.1 M, pH 7.2) and adjust the volume to 100 mL.

*S. epidermidis* ATCC 35984 (*S. epidermidis* 35984) was purchased from Guangdong Microbial Culture Collection Center (GDMCC) and stored at −80 °C. The frozen bacterial stock was inoculated into Trypticase Soy Broth (TSB, hopebio, China) and cultured at 37 °C with shaking at 180 r/min for 12 h. To prepare a fresh bacterial suspension, *S. epidermidis* 35984 was inoculated into TSB medium and cultured until the mid-log phase.

### Plant material and UPLC-MS/MS analysis

The *Rhizoma Coptidis* was purchased from Shanghai Leiyunshang Pharmaceutical Co., Ltd., and its origin is Sichuan, China.

To prepare the RCE, 50 g of powdered *Rhizoma Coptidis* was immersed in 500 mL of distilled water and boiled at 100 °C for 60 min, followed by filtration. The resulting filtrate was collected, and the residue was re-extracted with another 500 mL of distilled water under the same conditions. The final filtrate was concentrated to a dry powder using vacuum freeze-drying and stored at 4 °C.

The RCE was dissolved in acetonitrile, subjected to ultrasonic treatment (250 W, 40 kHz, 60 min) in an ice bath. Afterward, it was centrifuged at 20,000 × g at 4 °C for 10 min. UPLC-MS/MS analysis was performed following the method described by Li et al. [24], with slight modifications. The resulting supernatant was collected, filtered through a 0.22 μm membrane, and analyzed using UPLC-MS/MS Q-TOF with a Triple TOF 6600 + System (SCIEX, USA). Chromatographic separation was carried out on a Phenomenex Luna C18 column (250 mm × 4.6 mm, 5 μm). The mobile phases included acetonitrile (eluent A) and 0.1% aqueous formic acid (eluent B), with a linear gradient: 0–30 min, 10–90% A; 30–40 min, 90% A, at a flow rate of 1 mL/min. The injection volume was 5 µL, and the column temperature was maintained at 40 °C. Full scans were conducted in ESI positive ion mode within the m/z 50-1000 Da range. Data acquisition and processing were performed using SCIEX OS and MSDIAL V4.6 software.

### Evaluation of the antibacterial activity of RCE *in vitro*

The minimum inhibitory concentration (MIC) of RCE against *S. epidermidis* 35984 was determined using a broth microdilution method with some modifications according to the recommendations of CLSI M07-A8 by referring to the methods of Wiegand et al. [25] and Luz-Veiga et al. [26]. A two-fold serial dilution of the extract was prepared in TSB, and 100 µL of each concentration of the extract was added to each well, followed by the addition of 100 µL of *S. epidermidis* 35984 bacterial suspension (adjusted to 1 × 10^6^ CFU/mL). This resulted in final extract concentrations of 100, 50, 25, 12.50, 6.25, 3.125, 1.5625, 0.78125, and 0.390625 mg/mL, with a final bacterial concentration of 1 × 10^6^ CFU/mL in each well. Due to the natural color of the extract, a sample control group was also set, where 100 µL of each concentration of the extract was mixed with 100 µL of TSB medium. A growth control group was prepared by adding 100 µL of TSB medium and 100 µL of bacterial suspension to the well. Additionally, a sterility control group was set by adding 200 µL of TSB medium to the well. The MIC was determined as the lowest concentration with no optical turbidity.

The growth curve assay was performed based on a previously reported method [27], with slight modifications. RCE was dissolved in TSB to prepare a TSB medium containing the MIC of the extract. A negative control group was set using TSB medium without the extract. Bacterial suspensions were diluted to a concentration of 1 × 10^8^ CFU/mL, and 1% (v/v) of the suspension was inoculated into the experimental and control groups. The cultures were incubated at 37 °C with shaking at 180 r/min. At different time points, 200 µL samples were taken from each group, and the optical density at 600 nm (OD_600_) was measured using a Multiskan SkyHigh microplate reader (Thermo Scientific, USA).

The time-kill assay was performed based on a previously reported method [28], with slight modifications. *S. epidermidis* 35984 cells were centrifuged at 3000 × g for 10 min to remove the supernatant, then washed three times with PBS. The bacterial cells were subsequently resuspended in TSB medium containing either 0× or 1× MIC of the RCE, with a final bacterial concentration of 5 × 10^5^ CFU/mL. The inoculations were stirred and incubated at 37 °C. At 0, 2, 4, 8, 12 and 16 h, 100 µL samples from the cultures were serially diluted ten-fold with 0.85% NaCl and plated onto Trypticase Soy Agar (TSA, hopebio, China) plates. After 24 h of incubation, the number of surviving colonies was determined by counting the colonies on all plates.

### Enzyme activity

*S. epidermidis* 35984 in the logarithmic phase was resuspended to a final concentration of 10^7^ CFU/mL after being washed three times with PBS. RCE was subsequently added to the suspension at concentrations of 2 ×, 1 ×, and 1/2 × MIC. A control group without the extract was included. The suspension was incubated with shaking for 5 h. After incubation, bacterial cells were collected by low-speed centrifugation at 6500 × g, washed with PBS, and resuspended. The suspension was then processed using an ultrasonic cell disruptor (550 W, 3 s on and 5 s off for 30 min). The suspension was centrifuged at 6500 × g for 10 min to remove cell debris. The ATPase and succinate dehydrogenase (SDH) activities of the samples were measured using ATPase assay kits and SDH assay kits (Nanjing Jiancheng Bioengineering Institute, Jiangsu, China).

The alkaline phosphatase (AKP) activity was measured with slight modifications to a previously reported method [29]. *S. epidermidis* 35984, cultured to the logarithmic phase, was washed with PBS and adjusted to 10^7^ CFU/mL. The suspension was treated with RCE at concentrations of 2 ×, 1 ×, and 1/2 × MIC, along with a control group that received no extract. The suspension was incubated at 37 °C with shaking at 180 r/min. Samples were taken at 0, 2, 4, 6, and 8 h, and the supernatants were separated by centrifugation at 4000 × g for 10 min. AKP activity in the supernatant was measured using an AKP assay kit (Nanjing Jiancheng Bioengineering Institute, Jiangsu, China).

### Membrane potential

Membrane potential measurement was performed with slight modifications to a previously reported method [30]. *S. epidermidis* 35984, cultured to the logarithmic phase, was washed with PBS and adjusted to a concentration of 10^7^ CFU/mL. RCE was added to the suspension at final concentrations of 2 ×, 1 ×, and 1/2 × MIC, with a control group that did not contain the extract. The suspension was incubated with shaking for 5 h. After incubation, bacterial cells were collected by centrifugation at 6500 × g, washed with PBS, and resuspended. Rhodamine 123 was then added to the suspension at a final concentration of 2 µg/mL. The mixture was incubated in the dark at 37 °C for 30 min, followed by washing with PBS three times and resuspension. The fluorescence intensity of the bacterial suspension was measured using a SpectraMax M5 multifunctional microplate reader (Molecular Devices, USA) with an excitation wavelength of 480 nm and an emission wavelength of 530 nm.

### The leakage of nucleic acid and protein

The leakage of nucleic acid and protein from *S. epidermidis* was determined following the reported methods [29, 31] with a slight modification. *S. epidermidis* 35984, cultured to the logarithmic phase, was washed with PBS and adjusted to 10^7^ CFU/mL. The suspension was treated with RCE at concentrations of 2 ×, 1 ×, and 1/2 × MIC, along with a control group that received no extract. The suspension was incubated at 37 °C with shaking at 180 r/min. Samples were taken at 0, 2, 4, 6, and 8 h, and supernatant was harvested by centrifugation at 4000 × g for 10 min. The supernatant was then filtered through a 0.22 μm membrane. Protein leakage was quantified using a BCA protein assay kit (Beyotime, Shanghai, China), while nucleic acid leakage was determined by measuring absorbance at 260 nm with a 759 S UV-visible spectrophotometer (Jinghua Instruments, Shanghai, China).

### Microscopic observation

The microscopic observation of *S. epidermidis* biofilm was conducted based on previous method [28] with a slight modification. Biofilm formation of *S. epidermidis* was observed using scanning electron microscopy (SEM). *S. epidermidis* was incubated in 24-well chambered cover slides at 37 °C for 12 h with or without RCE at MIC. The formed biofilms were washed with sterile PBS, fixed with glutaraldehyde, and dehydrated using ethanol. The freeze-dried samples were then gold-coated and observed using a Gemini 300 scanning electron microscope (ZEISS, Oberkochen, Germany).

Biofilm biomass was assessed using confocal laser scanning microscopy (CLSM). *S. epidermidis* was incubated in 24-well chambered cover slides at 37 °C with RCE at 0 ×, 1/2 ×, or 1 × MIC, and then removed after 12 h and 24 h of incubation. After washing with PBS, the samples were stained with propidium iodide (PI) and SYTO 9 in the dark for 15 min. Following two PBS washes, the samples were observed using an FV1200 laser scanning confocal microscope (Olympus, Tokyo, Japan).

### Biofilm formation inhibition assay and mature biofilm eradication assay

The inhibitory effects of RCE on biofilm formation were evaluated using a crystal violet staining method, as previously described [32], with a slight modification. In a 96-well plate, 100 µL of *S. epidermidis* 35984 suspension (10^6^ CFU/mL) was mixed with equal volumes of RCE at final concentrations of 2 ×, 1 ×, 1/2 ×, 1/4 ×, and 1/8 × MIC. The mixture was incubated in a 37 °C incubator for 16–18 h. Cultures without the extract served as the positive control, while the medium without bacterial inoculum was used as the blank control. The biofilms in the 96-well plate were washed five times with saline, fixed with 95% methanol for 15 min, and air-dried. The biofilms were then stained with 0.01% crystal violet solution at 37 °C for 20 min, followed by air drying. The wells were washed with saline until the wash solution was colorless, and then dried again. The bound crystal violet was released using anhydrous ethanol, and the OD_590_ was measured. The results were given in biofilm formation inhibition percentage, calculated according to the following formula:

$$\begin{array}{c}\mathrm{biofilm}\;\mathrm{formation}\;\mathrm{inhibition}\;\left(\%\right)=\left({\mathrm{OD}}_{\mathrm{positive}\;\mathrm{control}}-{\mathrm{OD}}_{\mathrm{assay}}\right)/{\mathrm{OD}}_{\mathrm{positive}\;\mathrm{control}}\times100,\\{\mathrm{OD}}_{\mathrm{positive}\;\mathrm{control}}:\;{\mathrm{OD}}_{590}\;\mathrm{of}\;\mathrm{positive}\;\mathrm{control}\;\mathrm{group};{\mathrm{OD}}_{\mathrm{assay}}:\;{\mathrm{OD}}_{590}\;\mathrm{of}\;\mathrm{assay}\;\mathrm{groups}.\end{array}$$

For the mature biofilm eradication assay, 100 µL of *S. epidermidis* 35984 suspension (10^6^ CFU/mL) was mixed with an equal volume of TSB and incubated at 37 °C for 10 h to allow biofilm formation. The mature biofilms were then exposed to RCE at concentrations of 8 ×, 4 ×, 2 ×, 1 ×, and 1/2 × MIC. Cultures without the extract were used as the positive control, while the medium without bacterial inoculum served as the blank control. Biofilm formation was assessed using the crystal violet staining method. The results were expressed as the biofilm eradication, calculated using the following formula:

$$\begin{array}{c}\mathrm{biofilm}\;\mathrm{eradication}\;\left(\%\right)\;=\;\left({\mathrm{OD}}_{\mathrm{positive}\;\mathrm{control}}\;-\;{\mathrm{OD}}_{\mathrm{assay}}\right)\;/\;{\mathrm{OD}}_{\mathrm{positive}\;\mathrm{control}}\times100,\\{\mathrm{OD}}_{\mathrm{positive}\;\mathrm{control}}:\;{\mathrm{OD}}_{590}\;\mathrm{of}\;\mathrm{positive}\;\mathrm{control}\;\mathrm{group};\;{\mathrm{OD}}_{\mathrm{assay}}:\;{\mathrm{OD}}_{590}\;\mathrm{of}\;\mathrm{assay}\;\mathrm{groups}.\end{array}$$

### Preparation of samples for transcriptomic and metabolomic analyses

*S. epidermidis* 35984 was inoculated into TSB medium and cultured to the logarithmic phase, with the bacterial suspension adjusted to a concentration of 10^7^ CFU/mL. RCE was added to the culture to a final concentration of MIC, with medium without the extract serving as the control. Both experimental and control groups were incubated at 37 °C with shaking at 180 r/min for 5 h. Subsequently, bacterial cells were collected by centrifugation (4 °C, 8000 × g, 5 min) and washed three times with pre-chilled sterile PBS. The bacterial cells were rapidly frozen in liquid nitrogen and stored at −80 °C. Three independent biological replicates were used for transcriptomic analysis, while six independent biological replicates were used for metabolomic analysis in each group.

### Total RNA extraction, cDNA library Preparation and sequencing

RNA extraction from the bacterial cells was carried out using the CTAB method, followed by the removal of genomic DNA. Only RNA samples of high quality were used for library construction. Instead of poly(A) purification, rRNA depletion was performed using the RiboCop rRNA Depletion Kit for Mixed Bacterial Samples (Lexogen, USA). The mRNAs were fragmented into 200 nt pieces using a fragmentation buffer. Double-stranded cDNA synthesis was initiated with random hexamer primers (Illumina Inc., California, USA), incorporating dUTP in place of dTTP during the second strand synthesis. The resulting cDNA was processed with end-repair, phosphorylation, and ‘A’ base addition, according to Illumina’s library preparation protocol. The RNA-seq transcriptome library was constructed following the Illumina Stranded mRNA Prep (Illumina Inc., California, USA), and sequencing was performed using the Illumina NovaSeq 6000 platform.

Base calling was performed on sequencing image signals using CASAVA, which converted them into textual data, subsequently stored in FASTQ format as raw data. Fastp (v0.19.5) was employed to remove adaptor sequences, trim bases from the 5’ end that were not A, G, C, or T, and eliminate low-quality bases at the ends of reads (with a sequencing quality score below Q20). Reads with more than 10% ambiguous bases (N bases) or shorter than 25 bp after quality trimming were excluded. After these steps, clean data were retained. The clean reads were mapped to the reference genome (GCA_000011925.1) using Bowtie2 (v2.3.5). Gene expression levels were quantified using Transcripts Per Million (TPM). Differential expression between the control and RCE-treated groups was analyzed using DESeq2 (v1.24.0), with *P*-values adjusted via the Benjamini-Hochberg (BH) method to control the false discovery rate. The thresholds for selecting significant samples were: *P* < 0.05 and |log_2_(fold change (FC))| > 1.0. Functional annotations of the DEGs were performed using Gene Ontology (GO) and Kyoto Encyclopedia of Genes and Genomes (KEGG) enrichment analyses.

### Untargeted metabolomic analysis

A 50 mg solid sample and a 6 mm diameter grinding bead were placed into a 2 mL centrifuge tube. For metabolite extraction, 400 µL of extraction solution (methanol: water = 4:1, v: v) containing 0.02 mg/mL of L-2-chlorophenylalanine as internal standard was added. The mixture was then ground for 6 min at −10 °C and 50 Hz using a Wonbio-96c frozen tissue grinder (Shanghai Wanbo Biotechnology Co. Ltd., Shanghai, China), followed by low-temperature ultrasonic extraction for 30 min (5 °C, 40 kHz). The samples were incubated at −20 °C for 30 min, centrifuged at 13,000 ×g for 15 min (4 °C), and the supernatant was transferred to injection vials for UPLC-MS/MS analysis.

The LC-MS/MS analysis was performed following the method described by Kong et al. [33]. UPLC-MS/MS analysis was performed using a Thermo UHPLC-Q Exactive HF-X system equipped with an ACQUITY HSS T3 column (100 mm × 2.1 mm, 1.8 μm, Waters, USA). The mobile phases consisted of 0.1% formic acid in water: acetonitrile (95:5, v/v) as solvent A and 0.1% formic acid in acetonitrile: isopropanol: water (47.5:47.5, v/v) as solvent B. The flow rate was set to 0.40 mL/min, the column was maintained at 40 °C, and a 3 µL injection volume was used. Mass spectrometric data were collected with a Thermo UHPLC-Q Exactive HF-X Mass Spectrometer, employing electrospray ionization (ESI) in both positive and negative modes. Key conditions included an auxiliary gas heating temperature of 425 °C, a capillary temperature of 325 °C, sheath gas flow at 50 psi, auxiliary gas flow at 13 psi, ion-spray voltage floating (ISVF) at ± 3500 V, and a normalized collision energy (NCE) range of 20-40-60 eV for MS/MS. The detection range covered 70–1050 m/z.

### Quantitative real-time PCR (qRT-PCR)

The qRT-PCR was conducted to confirm the RNA-Seq results by analyzing the expression of 4 DEGs selected from various pathways. The gene-specific primers used in this study are listed in Table 1. The expression levels were normalized using 16 S rRNA as an internal standard. RNA extraction was performed as described above. The qPCR reaction was performed according to the procedure described by Shao et al. [34].

**Table 1:** The primer sequences used for RT-qPCR analysis genes

Gene	Primers (5’−3’)	
*sdhC*	Forward	GGGTTATTCCGATAGGTG
	Reverse	TTAGCAGTGAAAGCGATG
*ndh*	Forward	TACCATCAAGTCCCAACC
	Reverse	TATAGCGCCACAGAACAC
*sgtA*	Forward	ACATGCACTTACAGCCTCTG
	Reverse	TGGCACCGTTGTAGTCAT
*dagK*	Forward	TAATACCAATGGGCACCG
	Reverse	CGAATCCTTTAATGTAATACGC
16 S rRNA	Forward	ACTCCTACGGGAGGCAGCAG
	Reverse	GGACTACHVGGGTWTCTAAT

### Statistical analysis

Physiological experiment results are presented as the mean ± SD of three replicates. Significant differences between groups (*P* < 0.05) were assessed using one-way analysis of variance (ANOVA) followed by Tukey’s multiple comparison test in SPSS 22.0 (IBM, New York, USA).

## Results

### Chemical components determined by UPLC-MS/MS

The chemical composition of the RCE was analyzed using UPLC-MS/MS. A total of 26 compounds were identified. Detailed information on these compounds, including their chemical names, CAS numbers, retention times (RT), and relative abundances, is provided in Table 2. Among these, umbelliferone (12.37%), hexamethylquercetagetin (11.96%), cafestol (11.88%), curdione (7.35%), and bilobalide (5.32%) were identified as the most abundant compounds.

**Table 2:** Main chemical compounds in RCE

No.	Compounds	CAS numbers	RT (min)	Relative content (%)
1	14-Deoxy-11,12-didehydroandrographolide	42895-58-9	12.89063	4.04% ± 1.23%
2	Cafestol	469-83-0	10.7886	11.88% ± 3.11%
3	Cinnamoyl putrescin	4950-65-6	11.07008	4.15% ± 0.81%
4	Esculin	531-75-9	11.36973	0.60% ± 0.05%
5	Isocurcumenol	24063-71-6	10.55695	3.98% ± 0.78%
6	Bilobalide	33570-04-6	14.06205	5.32% ± 1.35%
7	Hydrocinchonine	485-65-4	12.57348	1.14% ± 0.12%
8	Vasicine	6159-55-3	13.04928	3.30% ± 1.65%
9	6-Methylflavone	29976-75-8	12.93595	2.16% ± 0.07%
10	Hydroquinidine	1435-55-8	11.74737	1.57% ± 0.36%
11	Hexamethylquercetagetin	1251-84-9	11.13808	11.96% ± 0.94%
12	Caffeine	58-08-2	10.1398	2.04% ± 0.25%
13	triacanthine	2365-40-4	14.58752	1.12% ± 0.32%
14	Lapachol	84-79-7	13.3666	4.21% ± 0.25%
15	Sophocarpine	6483-15-4/ 145572-44-7	11.30173	1.36% ± 0.69%
16	Curdione	13657-68-6	9.584434	7.35% ± 2.78%
17	Sophoridine	83148-91-8/ 6882-68-4	9.958983	4.76% ± 1.14%
18	Rhapontigenin	500-65-2	12.93595	1.51% ± 0.42%
19	Oxysophocarpine	26904-64-3	10.34313	2.28% ± 0.36%
20	Oxymatrine	16837-52-8	10.11732	2.33% ± 0.21%
21	Cinchonine	118-10-5	13.11728	4.24% ± 1.69%
22	Aspidocarpine	466-45-5	12.2285	1.73% ± 0.74%
23	Bufalin	465-21-4	12.05998	1.72% ± 0.08%
24	Asarone	2883-98-9	11.67937	1.06% ± 0.26%
25	Ajugasterone C	23044-80-6	9.7505	1.79% ± 0.69%
26	Umbelliferone	93-35-6	14.45868	12.37% ± 1.87%

### Effect of RCE on the growth of *S. epidermidis* 35984

**Fig. 1:**
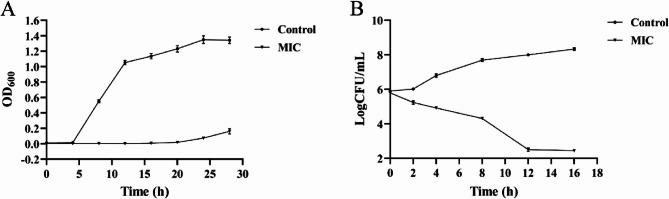
Growth curves (**A**) and time-kill curves (**B**) of *S. epidermidis* 35984 treated with RCE

The MIC of RCE against *S. epidermidis* 35984 was determined to be 6.25 mg/mL using the broth microdilution method. To further validate the inhibitory activity of RCE against *S. epidermidis* 35984, a growth curve of the bacterium was plotted. As shown in Fig. 1A, RCE significantly inhibited the growth of *S. epidermidis* 35984, compared to the control group. After treatment with the MIC of RCE, the OD_600_ of *S. epidermidis* 35984 remained below 0.02 for the first 20 h, increasing to 0.161 at 28 h. The time-kill curves of *S. epidermidis* 35984 were shown in Fig. 1B. The initial bacterial concentration was 5.89 LogCFU/mL, and after 12 h of treatment with the MIC of RCE, the bacterial concentration decreased to 2.51 LogCFU/mL. These results indicated that RCE exhibited strong inhibitory activity against *S. epidermidis* 35984.

### Effect of RCE on energy metabolism of *S. epidermidis* 35984

**Fig. 2:**
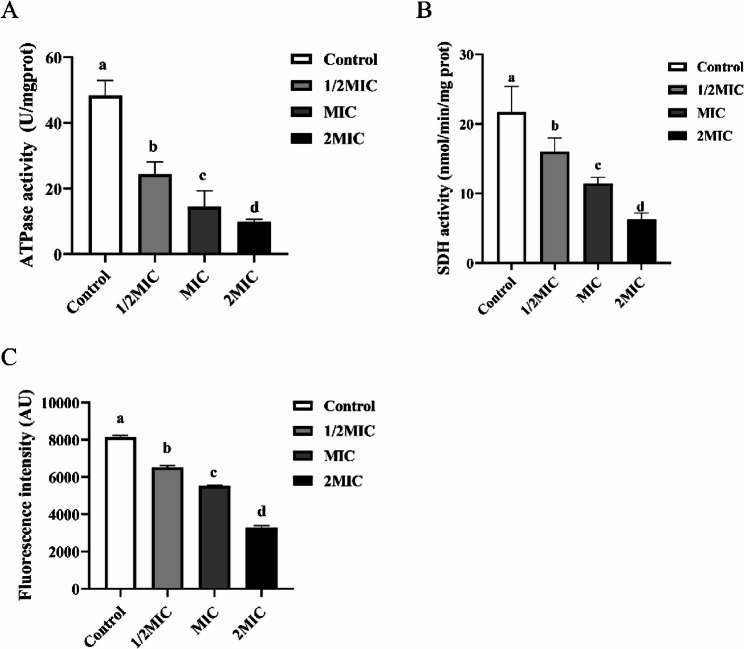
Effects of RCE on respiration and energy metabolism of *S. epidermidis* 35984. **A** The activity of ATPase. **B** The activity of SDH. **C** Membrane potential. Different letters in each figure indicate significant differences (*P* < 0.05)

ATPase plays a central role in energy metabolism and conversion, directly providing energy for biological activities [35]. Figure 2A showed the ATPase activity of *S. epidermidis* 35984 after treatment with different concentrations of RCE. The results indicated that the ATPase activity of *S. epidermidis* 35984 treated with RCE was significantly lower than that of the control group. Furthermore, higher concentrations of RCE corresponded to lower ATPase activity. Compared to the control group, the ATPase activity of *S. epidermidis* 35984 treated with the MIC of RCE decreased by 70.08%, while treatment with 2 × MIC resulted in a 79.67% reduction. These findings suggested that RCE effectively inhibited ATPase activity in *S. epidermidis* 35984, thereby impairing its energy supply.

SDH plays an essential role in primary metabolism of cells as the only enzyme that is involved both in the tricarboxylic acid cycle (TCA) and in respiration via the electron transport chain [36]. Disruption of its activity can impede the electron transfer between the TCA cycle and the respiratory chain, making SDH activity closely associated with cellular energy metabolism [37]. Figure 2B illustrated the effect of different concentrations of RCE on the SDH activity of *S. epidermidis* 35984. The SDH activity of *S. epidermidis* 35984 treated with 1/2 × MIC, 1 × MIC, and 2 × MIC of RCE were significantly lower than that of the control group. These results indicated that RCE damaged the TCA cycle and respiratory chain of *S. epidermidis* 35984 by inhibiting SDH activity, thereby disrupting normal energy metabolism and ultimately impairing the growth of *S. epidermidis* 35984.

The effect of RCE on the membrane potential of *S. epidermidis* 35984 was shown in Fig. 2C. Membrane potential refers to the electrical potential difference across the cell membrane, which is essential for ATP production and maintaining normal cellular functions [38]. Rhodamine 123, a lipophilic cationic dye, can penetrate the cell matrix through the transmembrane potential, and its fluorescence intensity reflects changes in membrane potential [39]. In untreated *S. epidermidis* 35984 cells, the fluorescence intensity was 8145.35 AU. After treatment with RCE at 1/2 × MIC, 1 × MIC, and 2 × MIC, the fluorescence intensity decreased to 6520.98 AU, 5531.05 AU, and 3266.20 AU, representing reductions of 19.9%, 32.1%, and 59.9%, respectively, compared to the control group. These reductions were statistically significant (*P* < 0.05). The significant decrease in membrane potential observed after treatment with RCE indicated depolarization of the cell membrane in *S. epidermidis* 35984. This depolarization likely disrupted ATPase activity and, consequently, energy metabolism in the bacterial cells.

### Effect of RCE on cell wall and cell membrane integrity of *S. epidermidis* 35984

**Fig. 3:**
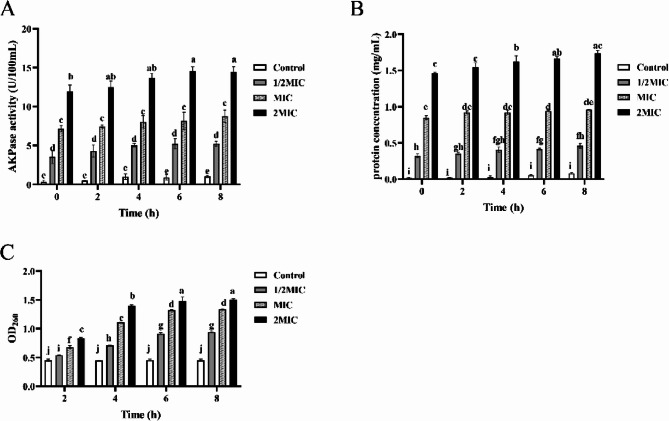
Effect of RCE on cell wall and cell membrane integrity of *S. epidermidis* 35984. **A** The activity of AKP. **B** The leakage of protein. **C** The leakage of nucleic acid. Different letters in each figure indicate significant differences (*P* < 0.05)

AKP is located between the bacterial cell wall and cell membrane, and its leakage occurs when the cell wall is damaged or its permeability increases [40]. The changes in AKP activity of *S. epidermidis* 35984 after treatment with different concentrations of RCE were shown in Fig. 3A. The AKP activity in untreated *S. epidermidis* 35984 cells showed minimal fluctuations over time. However, the AKP activity increased with prolonged treatment time and higher concentrations of RCE. These findings suggested that RCE disrupted the bacterial cell wall or enhanced its permeability, leading to the release of AKP.

The cell membrane is considered the critical barrier for bacterial viability, playing a vital role in maintaining cell morphology and the balance of materials and energy [41]. When bacteria are exposed to antimicrobial agents, damage to the cell membrane may result in the leakage of intracellular components, such as proteins and nucleic acids, into the extracellular environment [42]. The protein leakage from *S. epidermidis* 35984 was assessed using the BCA method, as shown in Fig. 3B. In the control group, the extracellular protein levels remained stable and were significantly lower than those in the bacterial suspensions treated with RCE. At 2 × MIC, the protein content in the bacterial suspension was significantly higher than that in the control and other treatment groups, indicating that RCE caused damage to *S. epidermidis* 35984 cells, increasing cell membrane permeability and leading to substantial leakage of intracellular proteins. Nucleic acids, as essential macromolecules found in all living cells, are considered fundamental structural units, and their leakage can result in cell death [43]. The leakage of nucleic acids from *S. epidermidis* 35984 following treatments with different concentrations of RCE was shown in Fig. 3C. In the control group, OD_260_ remained stable over time, whereas in the treated groups, OD_260_ values were significantly higher than in the control group. Specifically, the OD_260_ in the 2 × MIC treatment group rose to 1.477 within the first 6 h, which was 11.8% and 61.33% higher than in the MIC and 1/2 × MIC treatment groups at 6 h, respectively. After 6 h, the absorbance values in all treatment groups plateaued. These results indicated that treatment with RCE caused the leakage of intracellular nucleic acids and protein from *S. epidermidis* 35984 cells, with greater extract concentrations leading to increased leakage within a certain time frame.

### Effect of RCE on biofilm formation of *S. epidermidis* 35984

**Fig. 4:**
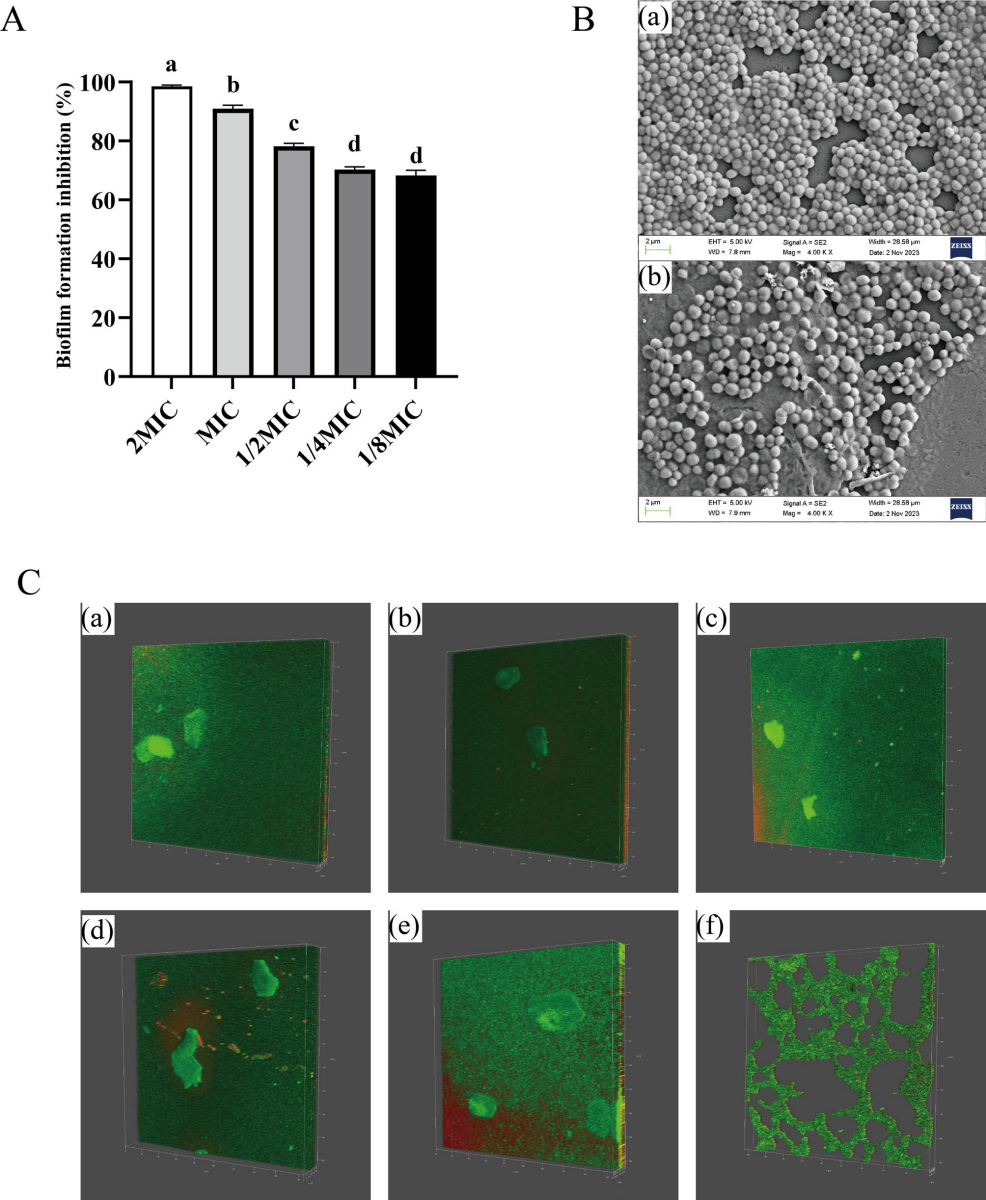
Inhibitory effect of RCE on biofilm formation of *S. epidermidis* 35984. **A** Inhibition of biofilm formation by *S. epidermidis* 35984 incubated for 16–18 h with RCE at concentrations of 1/8 × MIC, 1/4 × MIC, 1/2 × MIC, MIC, and 2 × MIC. Different letters in the figure indicated significant differences (*P* < 0.05). **B** SEM images of biofilms treated (**a**) without or (**b**) with RCE at MIC for 12 h. **C** CLSM images of biofilms treated with (**a**) 0 × MIC, (**b**) 1/2 × MIC, (**c**) MIC RCE for 12 h, and treated with (**d**) 0 × MIC, (**e**) 1/2 × MIC, (**f**) MIC RCE for 24 h

The inhibitory effect of different concentrations of RCE on the biofilm formation of *S. epidermidis* 35984 was evaluated using the crystal violet staining method. As shown in Fig. 4A, the biofilm formation inhibition at concentrations ranging from 1/8 × MIC to 2 × MIC ranged from 68.27% and 98.51%. The biofilm formation inhibition increased with the extract concentration, demonstrating a strong concentration-dependent effect. Notably, even at 1/8 × MIC, the inhibition exceeded 50%, indicating that RCE effectively inhibits biofilm formation in *S. epidermidis* 35984 in a concentration-dependent manner.

To investigate the inhibitory effect of RCE on biofilm formation, SEM and CLSM were used to observe the treated biofilms. The SEM results were shown in Fig. 4B. In the control group, *S. epidermidis* 35984 cells exhibited a tightly packed, regular spherical morphology with smooth edges and intact structures, with no noticeable gaps between cells (Fig. 4Ba). In contrast, after treatment with the MIC of RCE (Fig. 4Bb), the cells of *S. epidermidis* 35984 appeared shrunken, with irregular edges, and became loosely connected. Some cells showed ruptured membranes with intracellular contents leaking out. These observations indicated that treatment with the MIC of RCE caused structural deformation in *S. epidermidis* 35984 cells, leading to irreversible damage and demonstrating effective antibacterial activity. Previous study has found that treatment with alpha-mangostin significantly reduced biofilm aggregation and dispersal in *S. epidermidis* 35984, which is consistent with the results observed in this study [44].

The CLSM results were shown in Fig. 4C. PI can penetrate damaged membranes, bind to DNA, and emit red fluorescence, whereas SYTO 9 can penetrate both live and dead cells, bind to DNA, and emit green fluorescence [45]. The ratio of red to green fluorescence indicated the proportion of live and dead bacteria in the sample. In the control group, dense green fluorescence was observed (Fig. 4Ca and Cd), indicating thick and compact biofilms. After treatment with 1/2 × MIC of RCE for 24 h (Fig. 4Ce), the proportion of red fluorescence increased significantly, suggesting a higher percentage of dead bacteria and membrane damage induced by the extract. Following treatment with the MIC of RCE, the biofilm appeared sparse, loose, and noticeably thinner (Fig. 4Cc). After 24 h of incubation, the biofilm could no longer form an intact structure (Fig. 4Cf). These findings demonstrated that RCE effectively inhibited the biofilm formation of *S. epidermidi*s 35984.

### Eradication of mature biofilm of *S. epidermidis* 35984 by RCE

**Fig. 5:**
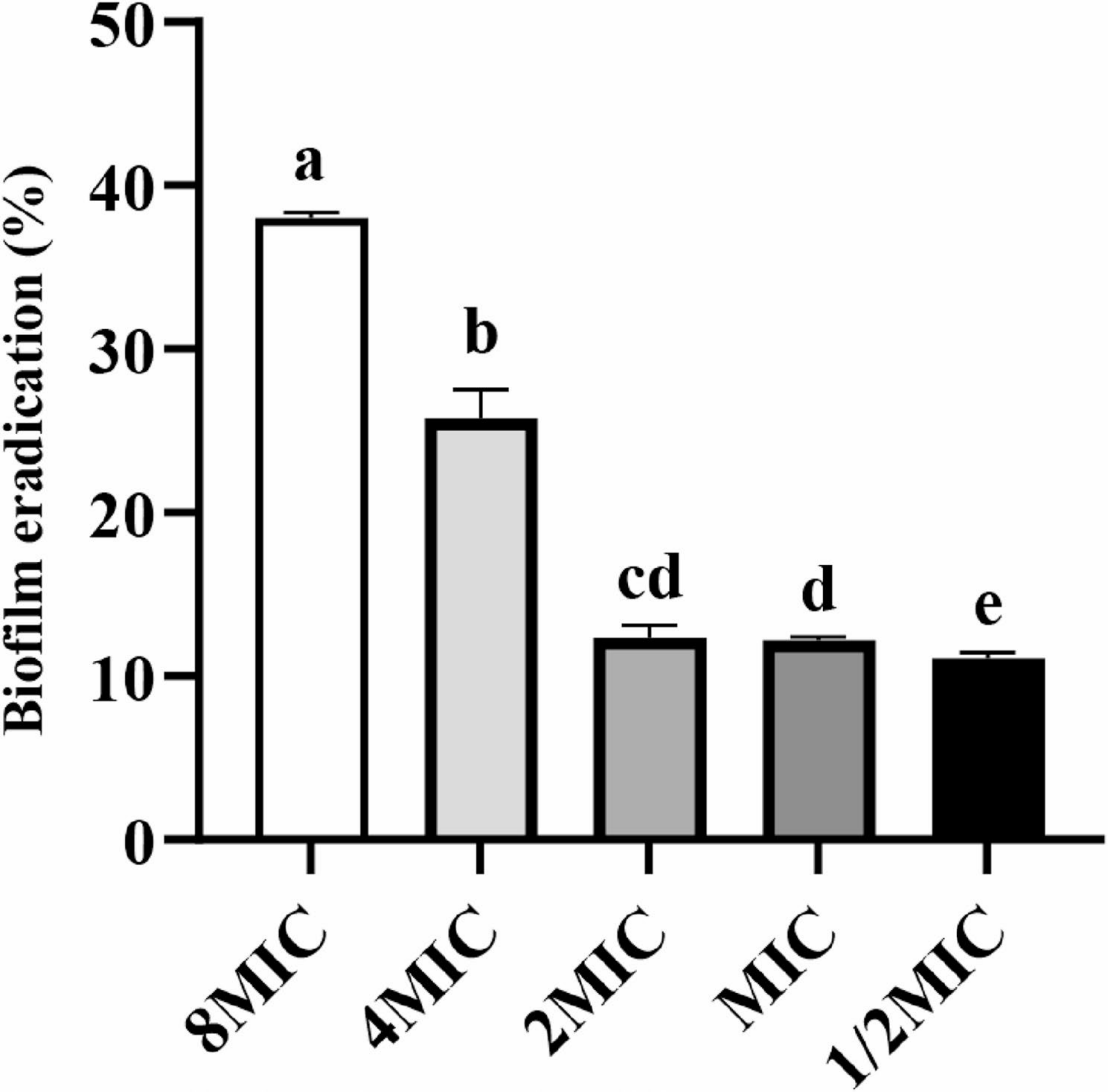
Biofilm eradication of RCE on the biofilm of *S. epidermidis* 35984. Different letters in the figure indicated significant differences (*P* < 0.05)

The effect of RCE at concentrations ranging from 1/2 × MIC to 8 × MIC on the eradication of mature biofilms formed by *S. epidermidis* 35984 was shown in Fig. 5. The biofilm eradication for 1/2 × MIC, 1 × MIC, and 2 × MIC were 12.36%, 12.19%, and 11.09%, respectively. However, when the extract concentration increased to 4 × MIC and 8 × MIC, the biofilm eradication rose to 25.75% and 38.02%, respectively. These results suggested that RCE partially eradicated mature *S. epidermidis* 35984 biofilms at higher concentrations.

### Global transcriptomic response

**Fig. 6:**
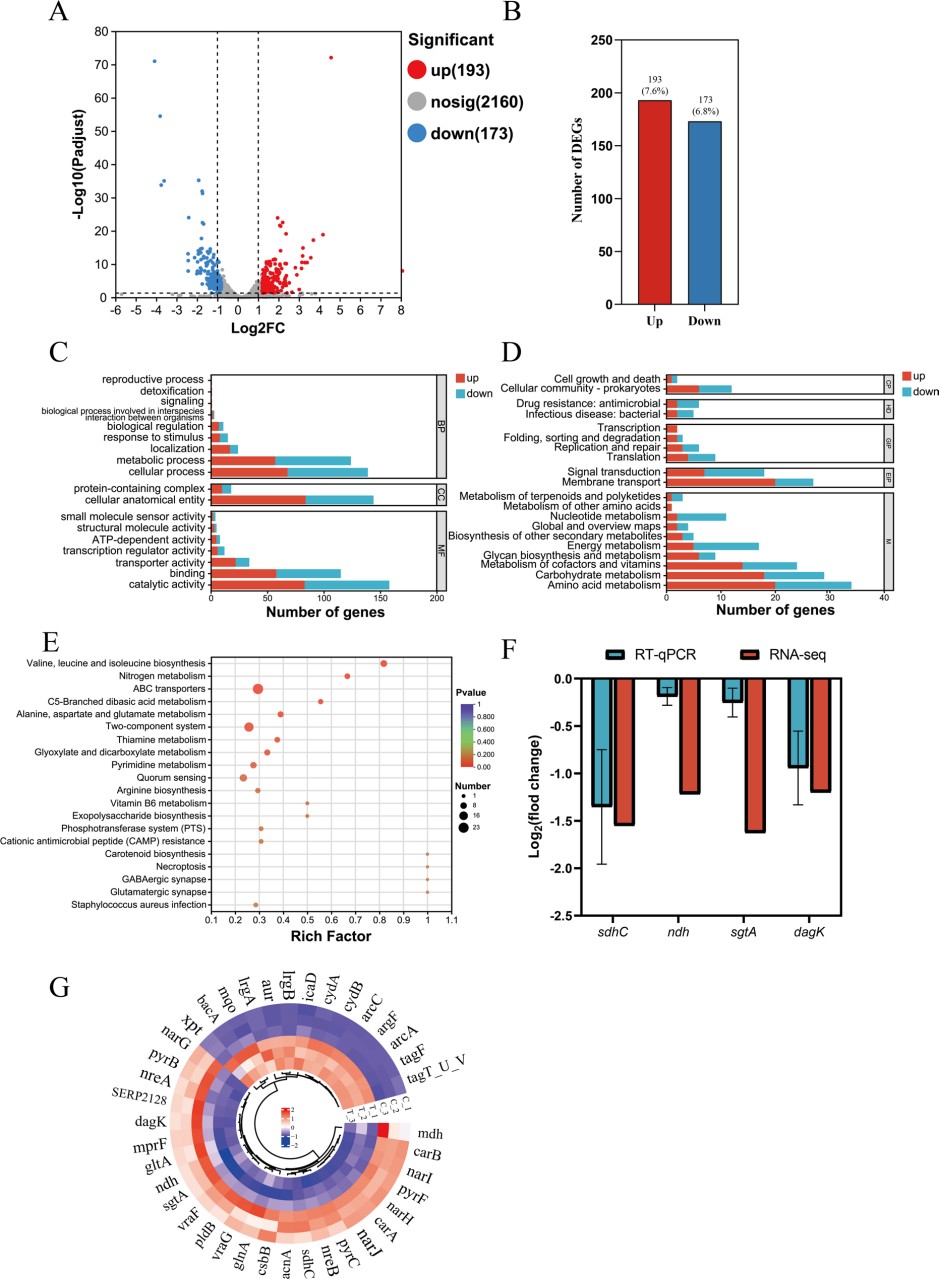
Global bioinformatics analysis of DEGs. **A** Volcano plot of DEGs treated with or without RCE at MIC with |log_2_FC| > 1.0 and *P* < 0.05. **B** The number of twofold DEG genes. **C** Histogram of GO annotation of DEGs treated with or without RCE at MIC. **D** Histogram of KEGG annotation of DEGs treated with or without RCE at MIC. **E** KEGG enrichment analysis of DEGs treated with or without RCE at MIC. **F** RT-qPCR analysis of the selected DEGs treated with or without RCE at MIC. RT-qPCR results were the mean values of -ΔΔC_T_ obtained from three biological replicates with error bars representing standard deviations. While all selected genes in RNA-seq data had *P* value of < 0.05, which meant significant difference. **G** Heatmap of DEGs in control and RCE-treated cells in selected metabolisms. T was treated with RCE, and C was used as a control

Genes with |log_2_FC| > 1.0 and *P* < 0.05 were identified as DEGs. A total of 366 DEGs were identified, including 193 upregulated and 173 downregulated, as shown in the volcano plot (Fig. 6A and B, Table S1).

The results of the GO analysis (Fig. 6C) showed that the DEGs identified after treatment with the MIC of RCE could be classified into three categories: biological process (BP), cellular component (CC), and molecular function (MF). In the BP category, metabolic process and cellular process were the main modules of DEG distribution. In the CC category, cellular anatomical entity was the predominant module. For MF, the most significant modules were catalytic activity and binding.

Functional annotation and pathway enrichment analysis were performed using the KEGG database. As shown in Fig. 6D, the DEGs were mainly annotated in pathways such as amino acid metabolism, carbohydrate metabolism, and metabolism of cofactors and vitamins. Additionally, membrane transport and signal transduction pathways were annotated with 20 and 7 upregulated DEGs, respectively. Other pathways included cellular community prokaryotes, glycan biosynthesis and metabolism, and energy metabolism, where upregulated DEGs were prominently represented. The downregulated DEGs are mainly annotated in pathways such as amino acid metabolism, energy metabolism, nucleotide metabolism, drug resistance: antimicrobial, translation, and replication and repair. KEGG enrichment analysis showed that RCE treatments at MIC resulted in DEGs associated with valine, leucine and isoleucine biosynthesis, ABC transporters, and nitrogen metabolism pathways (Figs. 6E). RT-qPCR was performed to validate the RNA-Seq data by analyzing the expression of DEGs in the same samples. Four DEGs (*sdhC*, *ndh*, *sgtA*, and *dagK*) were tested. The RT-qPCR results were largely consistent with the RNA-Seq data (Fig. 6F), indicating that the RNA-Seq results were reliable.

Following treatment with RCE, several DEGs associated with the synthesis of wall teichoic acid (WTA), such as *bacA*, *tagF*, and *tagT_U_V*, were upregulated, while DEGs related to the synthesis of lipoteichoic acid (LTA), such as *csbB*, were downregulated (Fig. 6G). Additionally, DEGs related to peptidoglycan synthesis, such as *sgtA*, and those involved in phosphatidic acid synthesis, such as *pldB* and *dagK*, were downregulated. These gene expression changes suggested that RCE disrupted the integrity of the cell wall and membrane. Some DEGs associated with arginine biosynthesis, such as *arcC*, *argF*, and *arcA*, were upregulated, while *glnA* was downregulated. Pyrimidine biosynthesis was also affected, with *carA*, *carB*, *pyrB*, *pyrC*, and *pyrF* downregulated. In terms of energy metabolism, most DEGs involved in the TCA cycle, including *sdhC*, *mdh*, *gltA*, and *acnA*, were downregulated, while DEGs encoding cytochrome bd oxidase, such as *cydA* and *cydB*, were upregulated. DEGs associated with antibiotic efflux, including *vraG* and *vraF*, were downregulated, while DEGs involved in the synthesis of polysaccharide intercellular adhesin (PIA), such as *icaD*, and those related to anti-autolysis factors, such as *lrgA* and *lrgB*, were upregulated.

### Global metabolomic response

**Fig. 7:**
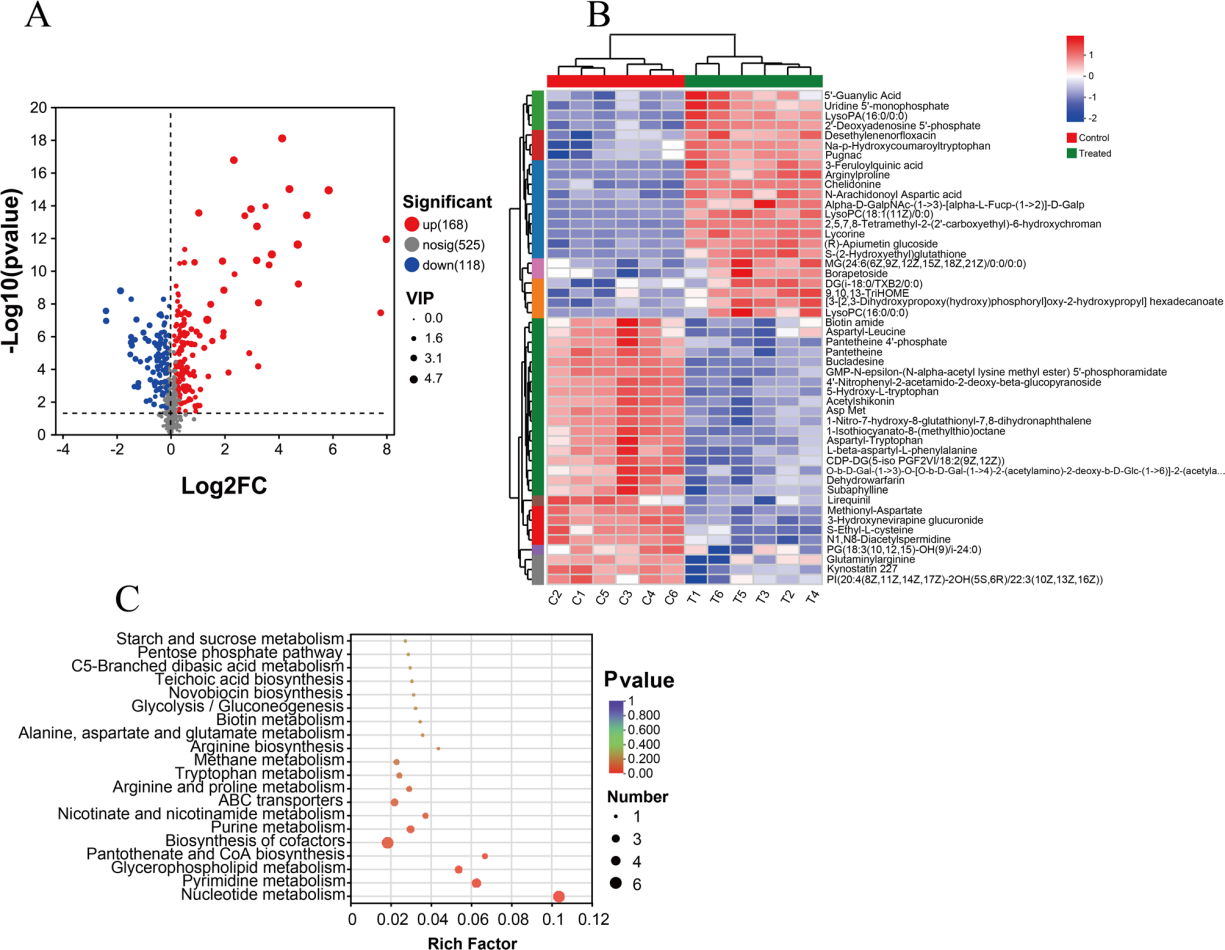
Global metabolic profiles analyses of *S. epidermidis* 3584 between RCE treatment at MIC and the control groups in positive and negative ion modes. **A** Volcano plot of DMs treated with or without RCE at MIC with VIP > 1, |log_2_FC| > 1 and *P* < 0.05. **B** Heatmap of DMs treated with or without RCE at MIC. **C** KEGG enrichment analysis of DMs treated with or without RCE at MIC

Based on the variable importance in projection (VIP > 1) from the PLS-DA model, combined with the criteria of |log_2_FC| > 1 and *P* < 0.05, a total of 109 and 177 significantly different metabolites (DMs) were identified under negative and positive ion modes, respectively. The results of the DMs were visualized using volcano plots, as shown in Fig. 7A and Table S2, with 168 metabolites significantly upregulated and 118 significantly downregulated. The heatmap of DMs (Fig. 7B) further confirmed that the relative abundance of metabolites in the RCE-treated group was significantly different from that in the control group. The KEGG metabolic pathway analysis (Fig. 7C) revealed that RCE primarily disrupted nucleotide metabolism, pyrimidine metabolism, glycerophospholipid metabolism, and purine metabolism in *S. epidermidis* 35984.

### Integrated analysis of transcriptomic and metabolomic results

**Fig. 8:**
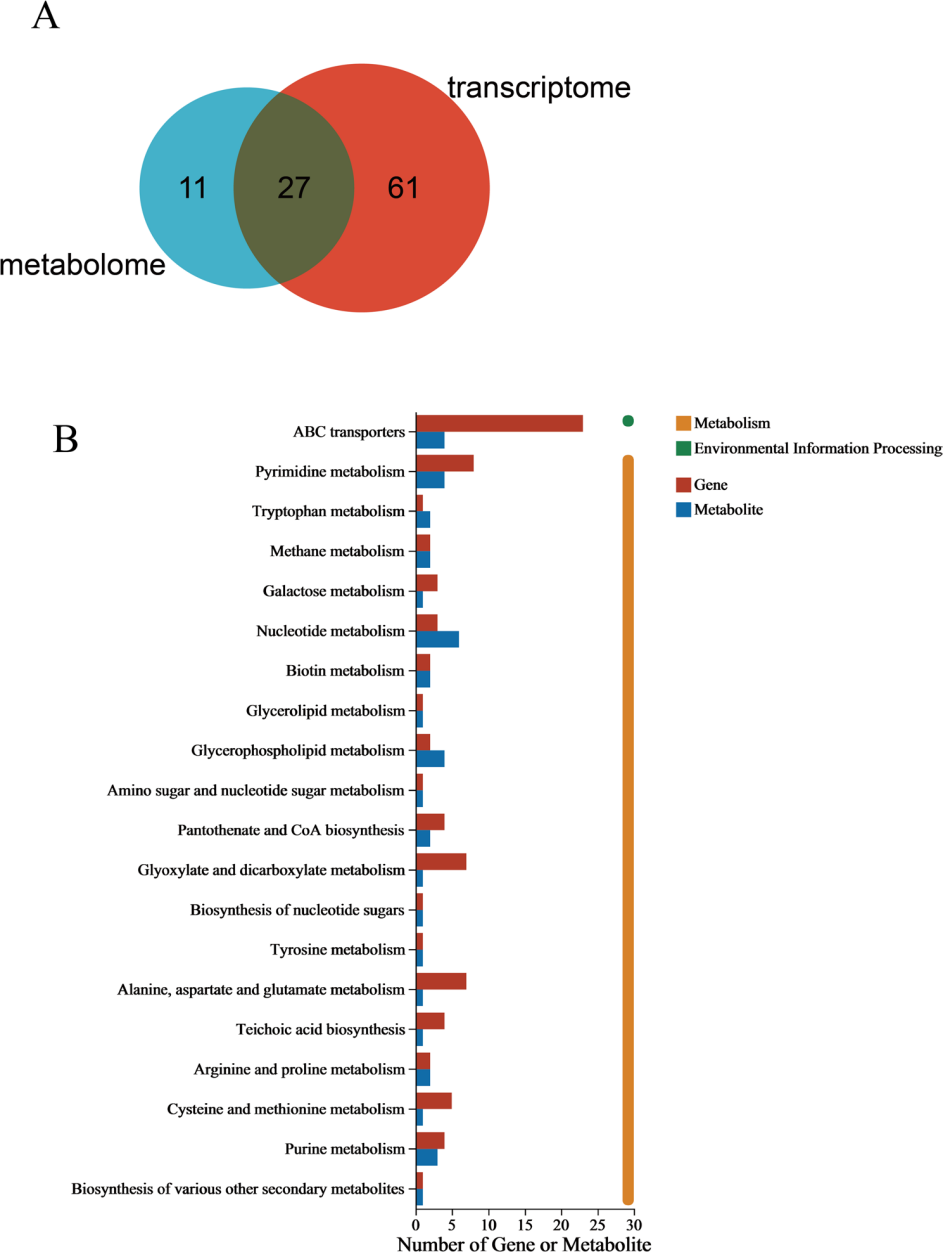
Integrated analysis of transcriptomic and metabolomic results of *S. epidermidis* 35984 treated with MIC of RCE. **A** Comparison of the number of pathways covered by DEGs and DMs. **B** Twenty of the pathways co-engaged by DEGs and DMs

To further elucidate the pathways through which RCE affects *S. epidermidis* 35984, an integrated analysis of DEGs and DMs was conducted. By comparing the pathways involving DEGs and DMs, 27 pathways were identified as being co-regulated by both the transcriptome and metabolome (Fig. 8A). The top 20 KEGG pathways with the highest overlap of DEGs and DEMs were visualized (Fig. 8B). These pathways were primarily associated with ABC transporters, pyrimidine metabolism, nucleotide metabolism, and glycerophospholipid metabolism.

## Discussion

*S. epidermidis* is a commensal bacterium found on the skin, which, when stressed or damaged, forms biofilms that can resist the bactericidal effects of antibiotics, promote self-protection mechanisms, and prevent damage from external factors. Biofilm formation is considered one of the most important virulence factors of *S. epidermidis*. Previous studies have reported the antimicrobial activity of RCE and berberine against various bacterial strains [17–19, 21]. However, most research on the antimicrobial properties of RCE has primarily focused on foodborne pathogens, with applications centered on food preservation and antimicrobial protection, while studies on its effects on skin microbiota remain limited. In this study, we analyzed the major components of RCE using UPLC-MS/MS and confirmed its specific antimicrobial activity against *S. epidermidis*, with a MIC of 6.25 mg/mL. Growth curve and time-kill assays revealed that RCE exhibited strong inhibitory activity against *S. epidermidis* 35984. We measured ATPase and SDH activities, as well as membrane potential, and found that RCE disrupted ATPase activity, the TCA cycle, and the respiratory chain, thereby impairing normal energy metabolism and ultimately inhibiting the growth of *S. epidermidis* 35984. Enzyme assays for AKP activity, as well as protein and nucleic acid leakage, indicated that RCE damaged the integrity of the cell membrane and wall. Additionally, RCE was shown to inhibit biofilm formation and, at higher concentrations, could eradicate mature biofilms. SEM and CLSM analyses further confirmed the biofilm-inhibitory effects of RCE. Through integrated transcriptomic and metabolomic analyses, we explored the antimicrobial activity and biofilm inhibition mechanisms of RCE against *S. epidermidis* 35984.

The bacterial cell wall and cell membrane play critical roles in maintaining the intrinsic morphology of bacteria, signal recognition, intracellular osmotic pressure, and the exchange of substances between the intracellular and extracellular environments [46]. Teichoic acids in the cell wall of Gram-positive bacteria perform multiple functions, including structural support, immune modulation, and enhancement of pathogenicity [47]. Among the cell wall-related DEGs, *bacA*, *tagF*, and *tagT_U_V* were significantly upregulated, while *csbB*, and *sgtA* were significantly downregulated. The genes *bacA*, *tagF*, and *tagT_U_V* encode undecaprenyl-diphosphatase, teichoic acid biosynthesis protein F, and polyisoprenyl-teichoic acid–peptidoglycan teichoic acid transferase, respectively, and are associated with the synthesis of WTA. Conversely, *csbB* is involved in the synthesis of LTA. The upregulation of WTA-related genes and the downregulation of LTA-related genes suggested that treatment with RCE prompted *S. epidermidis* 35984 to repair its cell wall by enhancing WTA synthesis to resist external stress, while reducing LTA synthesis may lower the sensitivity of lipid-associated structures. Peptidoglycan, a unique macromolecular component of bacterial cell walls, is essential for cell wall synthesis [46]. The *sgtA* gene encodes monofunctional glycosyltransferase, which catalyzes the glycosyl transfer reaction in peptidoglycan precursors, facilitating glycan chain elongation. The downregulation of *sgtA* indicated that peptidoglycan synthesis in *S. epidermidis* 35984 was inhibited. Phospholipids are essential components of the cell membrane, and phosphatidic acid serves as a critical precursor for phospholipid biosynthesis [48]. Following treatment with RCE, DEGs involved in phosphatidic acid synthesis, such as *pldB* and *dagK*, were significantly downregulated. These genes encode lysophospholipase and diacylglycerol kinase, respectively. Metabolomic analysis further revealed a significant reduction in CDP-diacylglycerol levels after treatment with RCE. These findings suggested that the extract inhibited the phospholipid biosynthesis pathway by targeting critical intermediates, thereby impairing the membrane repair system and exerting potent antibacterial effects. Overall, RCE disrupted the integrity of the cell wall and membrane in *S. epidermidis* 35984. It likely suppresses bacterial growth by reducing the synthesis of essential compounds vital for survival. This was consistent with biological assays, which demonstrated that treatment with RCE increased the permeability of the cell membrane and cell wall in *S. epidermidis* 35984, causing irreversible structural damage to the cells.

**Fig. 9:**
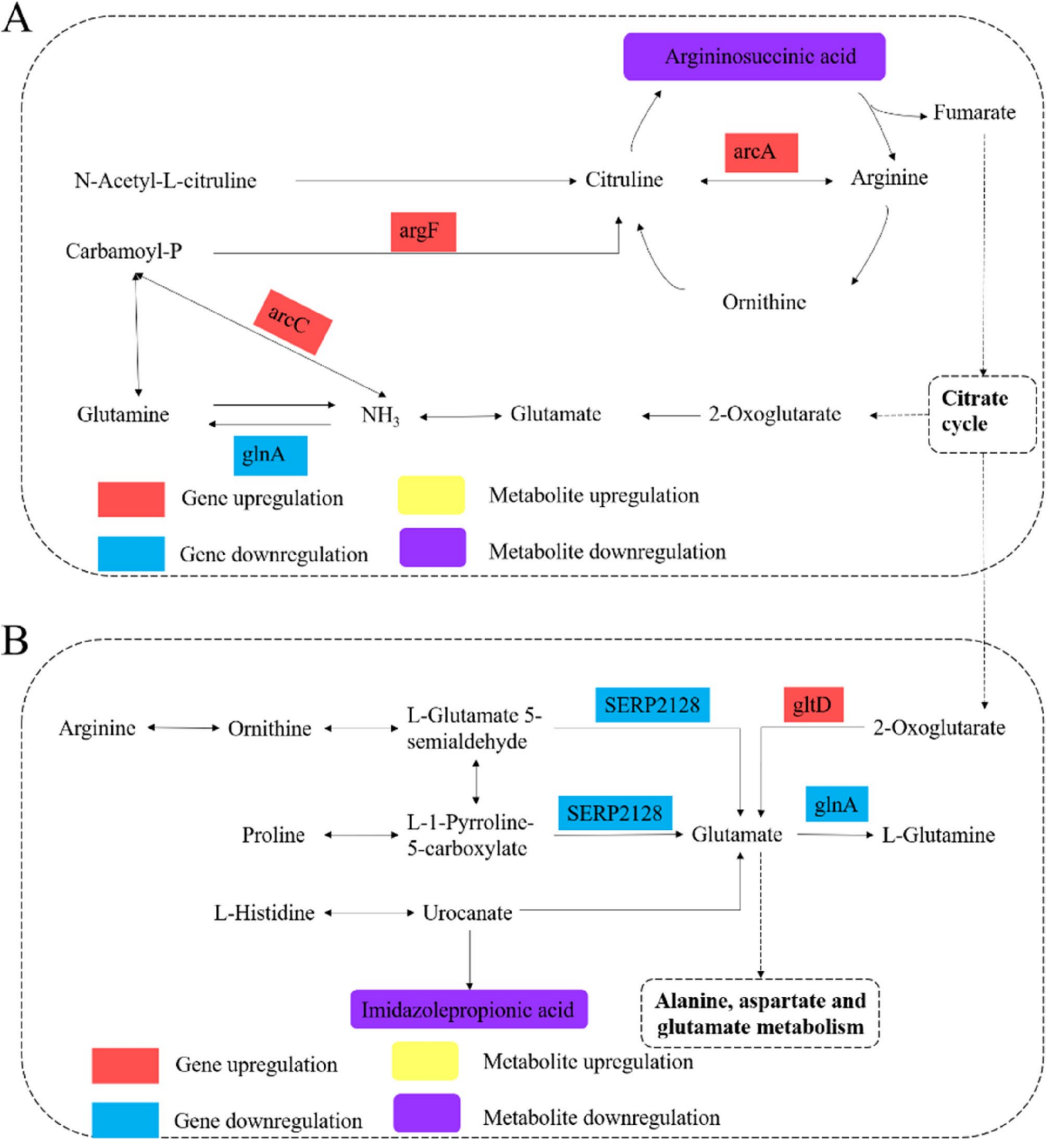
Pathway analysis of DEGs and DMs associated with Amino acid metabolism. **A** Arginine Biosynthesis. **B** Catabolism of arginine, proline, and histidine to glutamate

Amino acids, the fundamental building blocks of proteins, are key metabolites in bacterial biosynthesis and metabolic processes, playing an essential role in bacterial growth and metabolism [49]. Studies have shown that antimicrobial agents can disrupt amino acid synthesis and metabolism, leading to metabolic imbalances and inhibition of bacterial growth [50–52]. Arginine, one of the most versatile amino acids, participates in metabolic interconversion with proline and glutamate. It also serves as a precursor for the synthesis of proteins, nitric oxide, creatine, polyamines, guanidinoacetate, and urea [53]. Arginine metabolism is widespread in many microorganisms, enabling them to adapt to harsh environments and host defenses. In the arginine biosynthesis pathway (Fig. 9A), DEGs encoding carbamate kinase (*arcC*), ornithine carbamoyltransferase (*argF*), and arginine deiminase (*arcA*) were significantly upregulated, while *glnA*, encoding glutamine synthetase, was significantly downregulated. As argininosuccinic acid is a precursor for fumarate, which enters the TCA cycle, this suppression suggests an indirect impact on the TCA cycle. Figure 9B illustrated the degradation pathways of arginine, proline, and histidine to glutamate. The SERP2128 involved in arginine metabolism, proline metabolism, and was notably downregulated, along with a decrease in the content of imidazole propionate, indicating that the degradation of arginine, proline, and histidine to glutamate was inhibited. In summary, treatment of *S. epidermidis* 35984 with MIC of RCE disrupted amino acid metabolism, indicating that the extract disturbs the amino acid metabolic balance within the bacterium, thereby inhibiting its normal physiological activities.

**Fig. 10:**
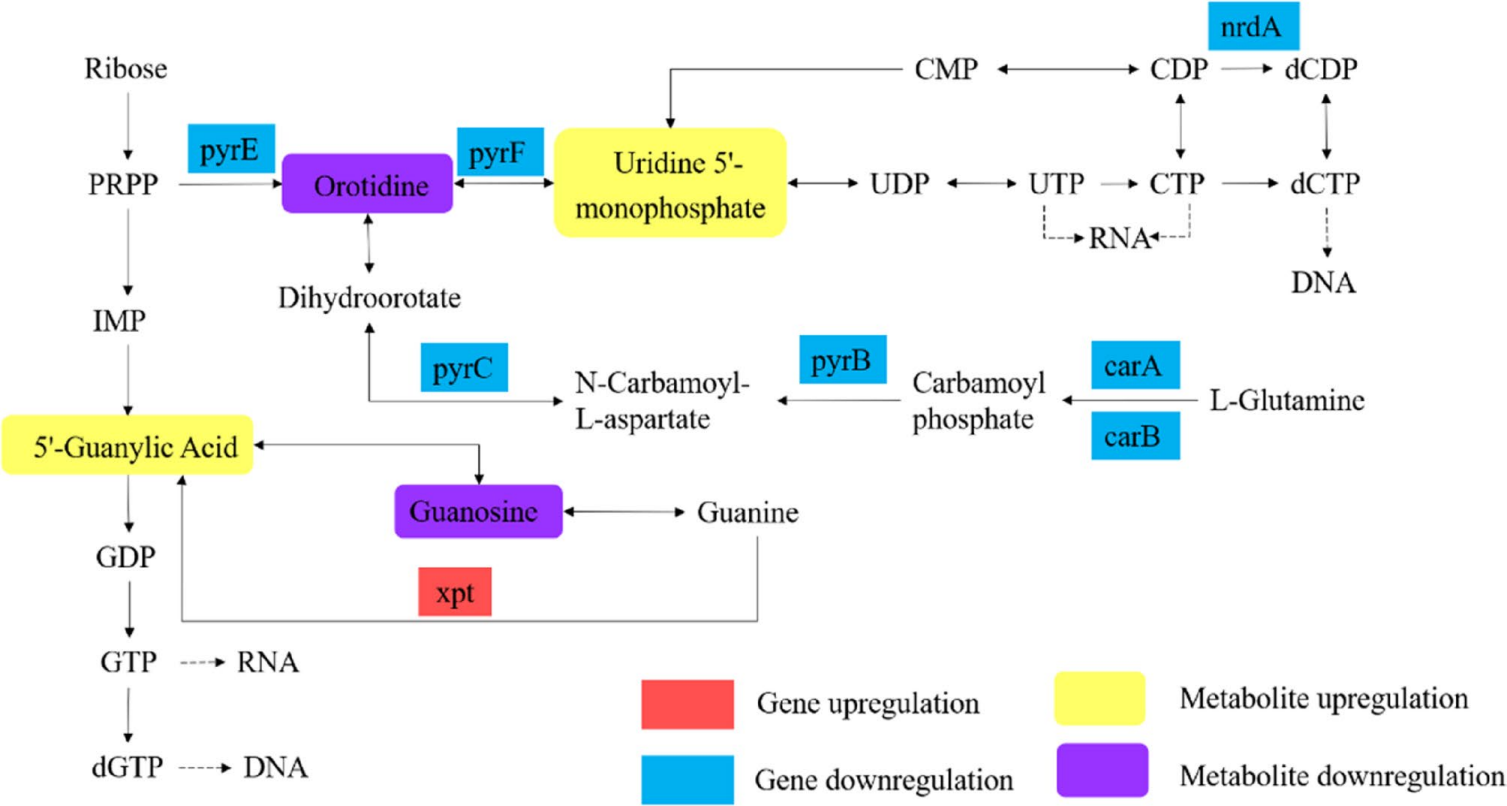
Pathway analysis of DEGs and DMs associated with nucleotide metabolism

Nucleotides are vital metabolites in all living organisms, functioning as substrates for RNA and DNA synthesis and serving as primary energy carriers for cellular processes. Additionally, nucleotides regulate diverse cellular activities, including metabolic processes and gene expression [54]. Figure 10 illustrated the pathway analysis of DEGs and DMs associated with nucleotide metabolism. The de novo pyrimidine biosynthesis pathway involves a series of enzymatic reactions: L-glutamine is converted to carbamoyl phosphate by carbamoyl-phosphate synthase (encoded by *carA* and *carB*), followed by the production of N-carbamoyl-L-aspartate through aspartate carbamoyltransferase (*pyrB*). N-carbamoyl-L-aspartate is subsequently converted into dihydroorotate via dihydroorotase (*pyrC*), which is further transformed into orotidine. Finally, orotidine is decarboxylated by orotidine-5’-phosphate decarboxylase (*pyrF*) to produce uridine 5’-monophosphate (UMP). The downregulation of *carA*, *carB*, *pyrB*, *pyrC*, and *pyrF*, along with reduced levels of orotidine, indicated that treatment with RCE inhibited the de novo pyrimidine biosynthesis pathway in *S. epidermidis* 35984. Interestingly, UMP levels were upregulated, suggesting a compensatory response to maintain UMP homeostasis. In the purine biosynthesis pathway, 5’-guanylic acid (GMP) plays a pivotal role in RNA and DNA synthesis. The observed downregulation of guanosine and upregulation of GMP suggest alterations in both the purine salvage and de novo synthesis pathways. Notably, the *xpt* gene, which encodes the enzyme responsible for converting guanine to GMP, was significantly upregulated. This finding highlighted a stress response aimed at sustaining purine nucleotide balance under the stress of RCE.

**Fig. 11:**
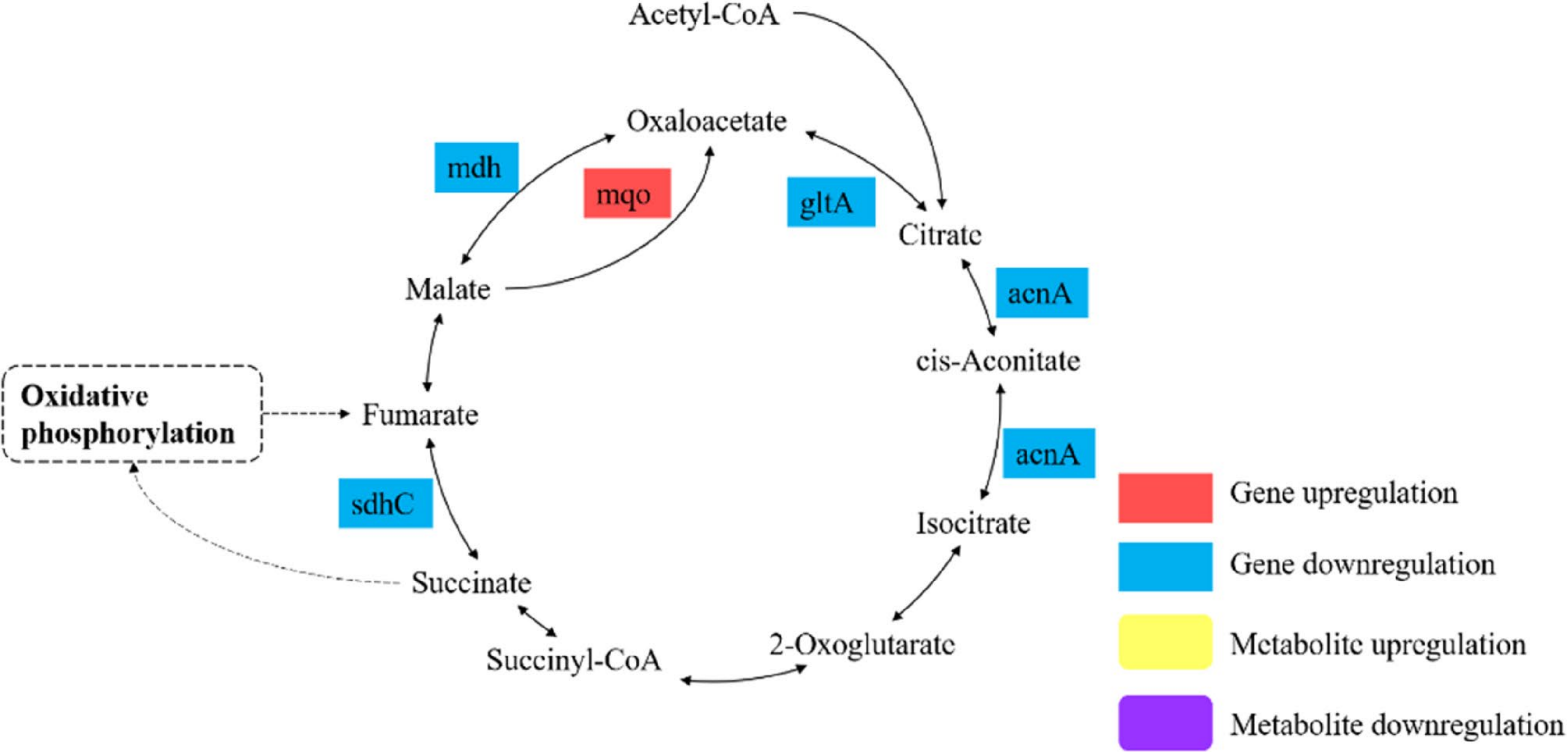
Pathway analysis of DEGs and DMs associated with energy metabolism

The TCA cycle is a critical pathway for cellular energy production and represents a core component of energy metabolism. In prokaryotes, the TCA cycle occurs within the cytoplasmic membrane and involves a series of enzyme-catalyzed reactions that oxidize organic molecules (such as metabolites derived from carbohydrates, fats, and proteins) to generate energy and metabolic intermediates. The TCA cycle is associated with the generation of reactive oxygen species (ROS) in *S. epidermidis* [55]. Figure 11 illustrated the pathway analysis of DEGs and DMs associated with energy metabolism. Key TCA cycle genes, including *sdhC*, *mdh*, *gltA*, and *acnA*, were significantly downregulated, indicating a suppression of TCA cycle activity following treatment with RCE. Conversely, the upregulation of *mqo*, which encodes malate: quinone oxidoreductase, suggests a compensatory mechanism to sustain oxaloacetate production via an alternative pathway that bypasses the conventional NADH-dependent reaction. This pathway employs quinone as the electron acceptor, potentially alleviating stress on the NAD⁺/NADH system, which may be disrupted by RCE. Enzymatic assays (Fig. 2B) further confirmed that SDH activity was significantly lower in the experimental group compared to the control group, demonstrating that RCE strongly inhibits SDH activity. Together, these findings indicated that *S. epidermidis* 35984 adapted to RCE by reducing reliance on the TCA cycle, and minimizing the production of ROS. The oxidative phosphorylation is the primary pathway cells use to obtain energy [56]. In oxidative phosphorylation, the genes *cydA* and *cydB*, encoding two key subunits of cytochrome bd oxidase, were upregulated. Cytochrome bd oxidase exhibits high oxygen affinity, allowing the maintenance of electron transport and energy metabolism under low-oxygen conditions [57]. It also has antioxidant functions, protecting cells from oxidative damage by breaking down reactive oxygen species such as superoxide (O₂⁻). Conversely, *sdhC*, encoding a critical transmembrane subunit of Complex II that links the TCA cycle with the electron transport chain, and *ndh*, encoding NADH dehydrogenase II, were downregulated. NADH dehydrogenase II facilitates electron transfer from NADH to coenzyme Q (CoQ), a key step in the electron transport chain [58]. The downregulation of *sdhC* and *ndh* suggested a reduction in TCA cycle activity and NADH oxidation, likely aimed at minimizing ROS generation and adapting to lower energy demands. The upregulation of *cydA* and *cydB*, combined with the downregulation of *sdhC* and *ndh*, indicated a shift in *S. epidermidis* 35984 from a highly efficient energy production mode (relying on Complex II and NADH dehydrogenase II) to a less efficient but more stress-tolerant mode involving cytochrome bd oxidase. This shift helped the bacterium maintain energy metabolism under adverse conditions induced by RCE.

The formation of *Staphylococcal* biofilm is a complex developmental process, which is divided into five sequential stages: (i) attachment, (ii) multiplication, (iii) exodus, (iv) maturation and (v) dispersal [59]. Treatment with RCE induced significant transcriptional changes in *S. epidermidis*, demonstrating a targeted effect on biofilm formation and regulatory pathways. The downregulation of genes associated with nitrate reductase activity (*nreA*, *nreB*, *narH*, *narG*, *narI*, and *narJ*) suggested a suppression of anaerobic respiration and nitrate reduction. This reduction likely minimized the production of reactive nitrogen species (RNS), such as nitric oxide (NO), which can destabilize biofilms through oxidative stress or NO-mediated biofilm dispersal [60]. Additionally, the downregulation of *vraG*, *vraF*, and *mprF*, responsible for antibiotic efflux and membrane modification, indicated decreased reliance on active resistance mechanisms. This may reflect a direct inhibitory effect of the extract on these energy-intensive pathways, potentially reducing the bacterium’s capacity to export antimicrobials. Conversely, the upregulation of *aur*, *icaD*, *lrgA*, and *lrgB* highlights enhanced structural regulation of the biofilm. The *icaD* is a key component of the intercellular adhesin polysaccharide synthesis system, playing a crucial role in extracellular polysaccharide production. It functions synergistically with *icaA* to catalyze the synthesis of PIA [61]. PIA, as the primary constituent of the biofilm matrix, is responsible for binding bacterial cells together and promoting their adherence to surfaces [62]. The increased expression of *icaD* suggested a compensatory increase in biofilm matrix production, reinforcing biofilm stability in response to extract-induced stress. Simultaneously, the upregulation of *lrgA* and *lrgB*, which encode anti-autolysis factors, indicated a reduction in autolysis and programmed cell death (PCD), preserving viable bacterial populations and supporting biofilm resilience. The upregulation of *aur*, encoding the metalloprotease aureolysin, emphasized its role in modulating biofilm structure. Aureolysin contributes to extracellular polymeric substance (EPS) turnover and potentially facilitates biofilm dispersal under nutrient-limited conditions by degrading specific matrix components. However, while these compensatory mechanisms may initially stabilize the biofilm, it is crucial to note that RCE also disrupts key biological processes that affect bacterial survival and biofilm formation. Previous study has shown that aqueous extracts of *Rhizoma Coptidis* also inhibited biofilm formation of *Streptococcus suis*. Specifically, at 1/4 × MIC (25 µg/mL) and 1/2 × MIC (50 µg/mL), significant inhibitory effects on biofilm formation were observed, with a concentration-dependent trend [19].

Our study suggested that RCE disrupted biofilm-associated resistance mechanisms while promoting structural adaptation. The induced upregulation of matrix-associated genes reflects a stress response to mitigate biofilm destabilization. However, this compensatory increase in biofilm matrix production didn’t counteract the broader effects of RCE on the bacterium’s metabolic and structural integrity. The extract’s action on multiple biological pathways—including energy metabolism, membrane integrity, and biofilm formation pathways—likely led to a reduction in *S. epidermidis* survival and its biofilm-forming capacity. This dual effect, where RCE both destabilized and stabilized biofilms through different mechanisms, demonstrated its potential as a biofilm-targeting agent. Targeting nitrate reduction pathways, efflux systems, and structural regulators could synergize with conventional antimicrobials to enhance therapeutic efficacy against biofilm formation by *S. epidermidis*.

## Conclusions

In conclusion, our study demonstrates that RCE exhibits potent antibacterial and anti-biofilm activity against *S. epidermidis* 35984. RCE treatment significantly impaired the energy metabolism, membrane integrity, and biofilm formation of *S. epidermidis*, as evidenced by reductions in ATPase and SDH activities, membrane depolarization, and leakage of intracellular components. Furthermore, transcriptomic and metabolomic analyses revealed significant alterations in genes and metabolites involved in amino acid metabolism, nucleotide synthesis, and energy metabolism, indicating that RCE disrupts key pathways necessary for *S. epidermidis* survival and biofilm formation. These findings provide new insights into the molecular mechanisms underlying the antibacterial and anti-biofilm properties of RCE and highlight its potential as an alternative therapeutic agent against *S. epidermidis*-associated infections, particularly those involving biofilm formation.

## Supplementary Information

Table S1: DEGs in *S. epidermidis *35984 treated with or without RCE; Table S2: DMs in *S. epidermidis* 35984 treated with or without RCE.

## Data Availability

RNA-seq data is available in the NCBI BioProject under accession number PRJNA1229122. The dataset can be accessed at: https://www.ncbi.nlm.nih.gov/sra/PRJNA1229122.

## Abbreviations

AKP: alkaline phosphatase
ATCC: American Type Culture Collection
BH: Benjamini-Hochberg method
BP: biological process
CC: cellular component
CLSM: confocal laser scanning microscopy
CoQ: coenzyme Q
DEGs: Differentially expressed genes
DMs: different metabolites
EPS: extracellular polymeric substance
ESI: electrospray ionization
FC: fold change
GDMCC: Guangdong Microbial Culture Collection Center
GMP: 5'-guanylic acid
GO: Gene Ontology
ISVF: ion-spray voltage floating
KEGG: Kyoto Encyclopedia of Genes and Genomes
LPS: lipopolysaccharide
LTA: lipoteichoic acid
MF: molecular function
MIC: minimum inhibitory concentration
N bases: ambiguous bases
NF-κB: nuclear factor kappa B
OD: optical density
PBS: phosphate-buffered saline
PCD: programmed cell death
PI: propidium iodide
PIA: polysaccharide intercellular adhesin
qRT-PCR: quantitative real-time PCR
RCE: *Rhizoma Coptidis* extract
RNS: reactive nitrogen species
RT: retention times
*S. epidermidis* 35984: *Staphylococcus epidermidis* ATCC 35984
SD: standard deviation
SDH: succinate dehydrogenase
SEM: scanning electron microscope
TCA cycle: tricarboxylic acid cycle
Th17: T-helper 17 cell
TPM: Transcripts Per Million
TSA: Trypticase Soy Agar
TSB: Trypticase Soy Broth
UMP: uridine 5'-monophosphate
UPLC-MS/MS: Ultra Performance Liquid Chromatography-Mass Spectrometry/Mass Spectrometry
VIP: variable importance in projection
WTA: wall teichoic acid
